# An Unusual Case of Hæmaturia

**Published:** 1883-03

**Authors:** P. R. Cortelyou

**Affiliations:** Marietta, Ga.


### AN UNUSUAL CASE OF HÆMATURIA.

By P. R. CORTELYOU, M.D., Marietta, Ga.

In August, 1881, I was called to see Mrs. ———, aged about thirty-five years, the mother of five children. She gave me the following history: Early in March, 1881, after a day of unusual exertion, and being on her feet a great deal, standing and walking, and having had to undergo more than ordinary exertion for several weeks previous, she noticed her urine was bloody on passing. This had continued from March until I was consulted. Upon investigation it was learned that, five years before, she had a similar attack, occurring about the sixth month of pregnancy, and troubling her until two or three months after her confinement, when it disappeared, and there was no return until the present attack. There was no pain whatever accompanying the hemorrhages, nor could any organic disease of the bladder, kidneys or urethra be discovered in the former attack; and a careful examination failed to reveal any special cause for the difficulty in the present attack.

The patient was placed on gallic acid, then tincture of the chloride of iron, ergot and various other recommended remedies, but without any effect on the bloody discharge. Rest in the recumbent position, which had been successful in the former attack, failed to give any relief this time. In September she became pregnant, which increased the difficulty, and towards the latter part of her pregnancy the urine assumed the consistency of jelly, with clots of coagulated blood.

The only thing complained of by the patient during the whole trouble was a weakness and increasing exhaustion. She was seen by several physicians at different times, but derived no benefit from treatment. In June, 1882, she was confined, and had an easy, natural labor, being delivered of a healthy male child, weighing six pounds. On the fourth day after confinement, the urine became normal, and has remained so ever since.

Among the ordinary causes enumerated by the textbooks are local lesions—as acute and chronic Bright's disease, active and passive congestion of the kidneys, calculus of the kidneys, uterus or bladder, cancer, tubercle and other morbid conditions of the bladder or prostate, supplementary or vicarious menstruation in rare cases, or from hemorrhoidal flux, or as a consequence of a paroxysm of spasmodic asthma. But the present case could not be traced to any of these causes.

The chief interest in this case lies in the complete absence of all symptoms of disease connected with the bloody urine; also the complete failure of all remedies to give any relief, and its sudden cessation soon after delivery.

